# *In Silico *discovery of transcription factors as potential diagnostic biomarkers of ovarian cancer

**DOI:** 10.1186/1752-0509-5-144

**Published:** 2011-09-19

**Authors:** Mandeep Kaur, Cameron R MacPherson, Sebastian Schmeier, Kothandaraman Narasimhan, Mahesh Choolani, Vladimir B Bajic

**Affiliations:** 1 Computational Bioscience Research Center, King Abdullah University of Science and Technology, Thuwal 23955-6900, Kingdom of Saudi Arabia; 2 Centre for Excellence in Genomic Medicine Research, King Abdul Aziz University, PO. Box 80216, Jeddah 21589, Kingdom of Saudi Arabia; 3 Diagnostic Biomarker Discovery Laboratory, Department of Obstetrics and Gynaecology, Yong Loo Lin School of Medicine, National University Health System, 5 Lower Kent Ridge Road, 119074, Singapore

## Abstract

**Background:**

Our study focuses on identifying potential biomarkers for diagnosis and early detection of ovarian cancer (OC) through the study of transcription regulation of genes affected by estrogen hormone.

**Results:**

The results are based on a set of 323 experimentally validated OC-associated genes compiled from several databases, and their subset controlled by estrogen. For these two gene sets we computationally determined transcription factors (TFs) that putatively regulate transcription initiation. We ranked these TFs based on the number of genes they are likely to control. In this way, we selected 17 top-ranked TFs as potential key regulators and thus possible biomarkers for a set of 323 OC-associated genes. For 77 estrogen controlled genes from this set we identified three unique TFs as potential biomarkers.

**Conclusions:**

We introduced a new methodology to identify potential diagnostic biomarkers for OC. This report is the first bioinformatics study that explores multiple transcriptional regulators of OC-associated genes as potential diagnostic biomarkers in connection with estrogen responsiveness. We show that 64% of TF biomarkers identified in our study are validated based on real-time data from microarray expression studies. As an illustration, our method could identify CP2 that in combination with CA125 has been reported to be sensitive in diagnosing ovarian tumors.

## Background

Ovarian cancer (OC) is the leading cause of death among gynecological malignancies and represents the fifth leading cause of cancer-related deaths in women. The disease is diagnosed at stage when cancer has already metastasized beyond the ovary in approximately 70% of patients and only 30% of these patients with this advanced-stage OC survive 5 years after initial diagnosis [1]. This inability to detect ovarian carcinoma during the early organ-confined stage combined with the lack of effective therapies for advanced-stage disease contributes to lethal effects of this cancer. In patients with metastasized OC, most relapse and ultimately die due to the development of drug resistance [2].

Early diagnosis greatly enhances the chances of successful cancer treatment. To this date, very few early-detection approaches have shown promise for routine clinical use. The most commonly used marker of OC is CA125, but it is expressed in 50-60% of patients during early stages of the disease [3]. Several biomarkers either individually or in combination with CA 125 have been proposed for early-detection and screening of OC [4]. FDA recently cleared an *In Vitro *Diagnostic Multivariate Index Assay (IVDMIA) i.e. OVA1 test that involves analysis of five serum biomarkers for assessing ovarian cancer risk in women [5]. Over the past few years it has become increasingly evident that many molecular changes observed in cancer cells involve deregulation of gene expression. Understanding the underlying molecular mechanisms of gene regulation could thus be crucial for identifying the key genes or proteins that can be exploited as prognostic or diagnostic biomarkers in OC. This makes transcription factors (TFs) an interesting target for further exploration in this direction [6]. The majority of oncogenic signaling pathways converge on sets of TFs that ultimately control gene expression patterns characteristic for tumor formation and progression, as well as metastasis. Since many of these TFs are inactive in the cancer affected tissues under normal physiological conditions and their expression and activities are tightly regulated, these TFs represent highly desirable and logical points of therapeutic interference in cancer development, progression and prognostication [7-9], markers for cancer [10], potential prognostic markers [7,11] and targets for drug therapy [12]. More recently the use of TFs as markers for the disease itself has been reported and they have been detected in the blood [10,13,14]. Another study [7] investigated the role of survival-related profile, pathways, and TFs in OC. The study reported that 13 out of 111 TFs were associated with overall survival in patients with OC. Since hormones also play an important role in gene expression [15] and are implicated in many cancers [16-19], it also becomes important to study the effects of hormones in cancers. It is documented that high levels of expression of estrogen receptor alpha (ERα) has been observed in many OCs and OC cells are growth responsive to both estrogen and anti-estrogens [20]. This emphasizes that ERα could have therapeutic potential for at least a sub-group of OC patients [21-23]. The hormone replacement therapy has also been linked with an increased risk of OC [24]. Recently, the prognostic value of estrogen receptors (ERs) for OC has been emphasized [25]. It is worth noting that the studies explaining the role of hormones in OC are few and require elaborative investigations [26]. Therefore, in the present study we focus on transcription regulation and also estrogen control of genes in OC. The question we addressed is to identify potential diagnostic TFs implicated in regulating the expression of OC genes that in turn could potentially be regulated by estrogen hormone. We aimed at linking hormone induction to overall transcription regulation of genes in cancer cells. We anticipate that the target pool of biomarkers could be revealed by studying specific signatures within the hormone dependent regulatory gene networks.

Computational approaches are a pragmatic and inexpensive way to identify the key regulatory genes, hence, pinpointing the targets for experimental validation. For example, bioinformatics methods were employed to identify candidate genes for discriminating different tumoral histotypes for OC, lung and breast cancer diagnostics [27]. In general, steroid hormones are recognized initially by hormone receptors that then bind to Hormone-Response Elements (HREs) on DNA and control the expression of some of their target genes. This can be used to predict a part of genes that are potentially controlled by hormone receptors under the condition that promoters of these genes contain HREs. Thus, the identification and characterization of HREs is critical for our understanding of hormone driven gene expression and regulation in various cancers and computational methods could be of great help [28-31]. To contribute to the discovery of diagnostic biomarkers for OC we developed a computational method which, in combination with the manual curation of the literature information, helped us to identify the potential biomarkers that could regulate a set of genes implicated in OC. Our analysis singles out several such TFs. The previous efforts that used the bioinformatics approach to identify a TF, E2F5, and then experimentally confirm it as a potential diagnostic biomarker for OC were made by our group [6]. Our current study is, to the best of our knowledge, the only one that targets through purely bioinformatics approach, transcriptional regulators of OC-associated genes as potential biomarkers in relation to estrogen responsiveness.

## Results and Discussion

This study identified potential biomarkers important for overall transcriptional regulation of OC genes and specifically for a sub-group of OC genes controlled by estrogen. To identify genes controlled by estrogen, two approaches were used: (a) prediction of estrogen response elements (EREs) on the promoters of OC genes, and (b) finding the experimental evidence for estrogen control in published databases. The presence of EREs in a promoter suggests that ERE sites may be used by activated hormone receptors and consequently could affect gene expression. The EREs were predicted on 246 promoters corresponding to 65 genes. Out of these 65 genes, 11 genes had experimental evidence of being responsive to estrogen as these genes were found in either KBERG [32] or ERTargetDB [33] databases. In the dataset, there were 258 genes lacking predicted EREs in their promoters. Out of these 258 genes, 66 were under estrogen control as supported by KBERG or ERTargetDB. We split the genes into four groups that were analyzed further. These four groups were:

Group 1) genes with predicted EREs and with experimental evidence of estrogen responsiveness (11 genes);

Group 2) genes with predicted EREs with no experimental evidence of estrogen responsiveness (54 genes);

Group 3) genes without ERE predictions but with experimental evidence of estrogen responsiveness (66 genes); and

Group 4) genes without ERE predictions and no experimental evidence of estrogen responsiveness (192 genes).

It is to be emphasized here that ERs can also affect gene expression by forming protein-protein complexes with other TFs such as activator protein-1 (AP-1), Sp1 family TFs, nerve factor-ß (*NF-ß*), etc. These complexes in turn can bind to the genes' promoters and regulate gene expression. Through these ERE-independent pathways, ERs can control the expression of many genes, making them estrogen-responsive, but without involvement of the full ERE. Therefore, the EREs, though good indicators that genes may be controlled by estrogen, are not essential for responsiveness to estrogen. On its own, the presence of EREs in the promoter region of a gene is not conclusive evidence of the hormonal control of expression of that gene. However, the presence of EREs in a promoter suggests that such ERE sites could be used by activated hormone receptors and consequently could affect gene expression.

### (a) Functional analysis of target genes

The GO analysis was performed as described in Material and Methods. The results for each group are discussed below:

Group 1: This group contains genes with predicted EREs and with experimental evidence of estrogen responsiveness. This group contains only 11 genes (3% of total 323 genes under study). The GO analyses of these genes show that 45% of genes in group 1 had kinase activity (additional file 1).

Group 2: The genes in this group (17% of 323 genes) have predicted EREs but they lack experimental evidence of being responsive to estrogen. It may be assumed that these genes might be under the control of estrogen hormone but this observation has not been conclusively demonstrated or supported by literature. GO analyses revealed that 24% (13 out of 54) had kinase activity and 26% of genes were involved in nucleotide binding (additional file 1).

Group 3: All the 66 genes (20%) in this group have no predicted EREs but have experimental evidence of being estrogen responsive. The genes in this group were mainly involved in organ development (32%), positive regulation of cellular processes (29%) and intracellular signaling cascade (26%). Only three (5%) were involved in regulation of I-kappaB kinase/NF-kappaB cascade (additional file 1).

Group 4: The genes in this group contain no ERE predictions, and have no experimental evidence in support of an estrogen response and constitute approximately 60% (192 of 323) of the total number of genes under study. Most of these genes (38%) were involved in cellular protein metabolic process. Some of the genes in this group (23%) were part of cell surface receptor linked signal transduction, while others (21%) had kinase activity (additional file 1).

### (b) Analysis of TF binding sites (TFBSs)

The gene groups were then subjected to TFBS analysis as explained in Materials and Methods. The results of TFBS analysis are summarized in following sections:

#### (i) Distribution of TFBSs in promoters of OC genes

**Figure 1:**
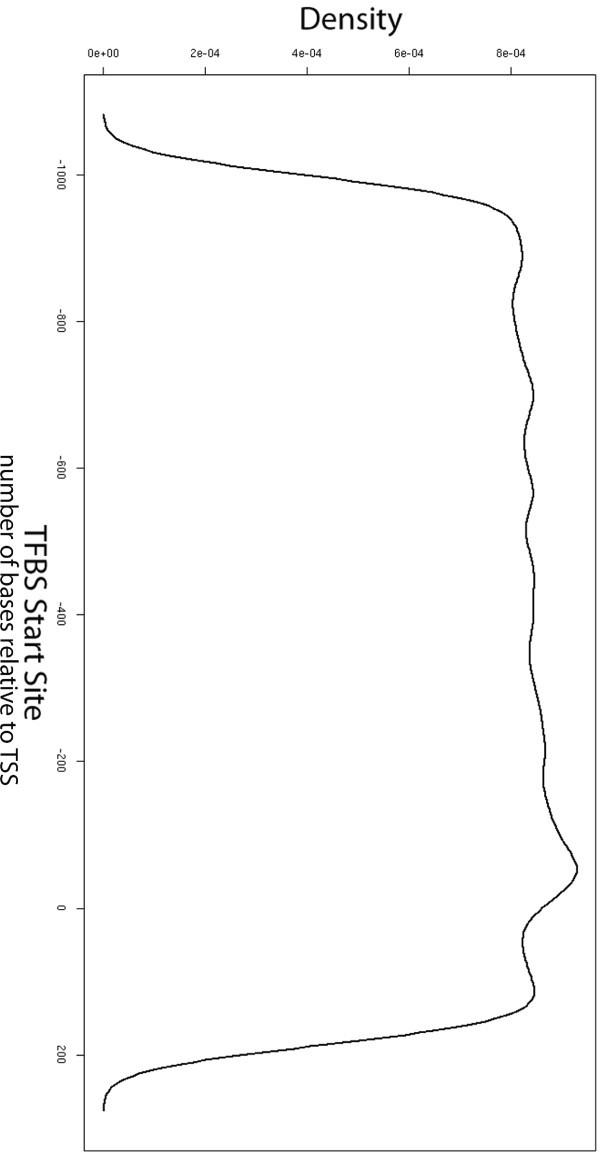
**Distribution of all predicted TFBSs in OC genes**. X-axis: distance in nucleotides from TFBS relative to TSS. Y-axis: estimated density of TFBSs as calculated by a Gaussian-kernel density function implemented in the R-statistical environment as "plot.density"; the distances of all TFBSs from the TSS are distributed across 512 points and convolved using a Gaussian kernel.

We predicted 9246 TFBSs corresponding to 299 TFs using 522 TRANSFAC matrices. The distribution of all predicted TFBSs in OC genes is presented in Figure 1. This figure shows the distribution of TFBSs using the Gaussian-kernel density estimator implemented in the R statistical environment under the function name, "plot.density"; the greatest density is just upstream of transcription start sites (TSSs).

#### (ii) Distribution of TFBSs and EREs in promoters of genes that contain predicted EREs

**Figure 2:**
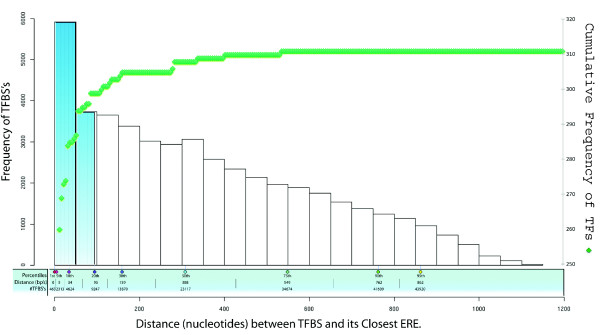
**Histogram and cumulative plot of distances between TFBSs and EREs in OC promoters**. X-axis: distance in nucleotides between TFBSs and its' closest ERE; maximum distance is limited to the size of promoters (1200 nt); frequencies of TFBSs for selected percentiles are given. Y-axis (left, histogram): frequency of TFBSs per 50 nucleotides (24 bins); the 20th percentile is highlight blue for convenience. Y-axis (right, dot-plot): cumulative frequency of TFBSs.

It has been demonstrated in the literature [34] that distance between ERE and TFBS is crucial for transcription of the gene. Some studies also suggest that most of the ERE effects are at very large distance from TSSs of genes they regulate [35]. The activities of the downstream promoter of vitellogenin gene A1 are shown to be controlled by EREs located 330 bases upstream of the TSS and it is speculated that EREs located up to 1.5 kb downstream can also control the promoter [36]. The distribution of distances between TFBSs and EREs within the promoters of OC genes that have predicted EREs is presented in Figure 2. For this analysis we pooled the results of group 1 and group 3 genes as both these groups had genes experimentally validated to be estrogen responsive. We found 260 TFs had TFBSs completely overlapping with EREs, whereas 299 TFs had TFBSs predicted at a maximum distance of 95 bases from predicted EREs. The detailed results are provided (additional file 2). Most of the TFs having TFBSs within a distance of 95 bases from predicted EREs were involved in cancer related biological processes. This suggests that the closeness of ERE to some other TFBSs implicated in our analysis could synergistically function in OC.

#### (iii) Identification of TFs unique to groups of genes

**Figure 3:**
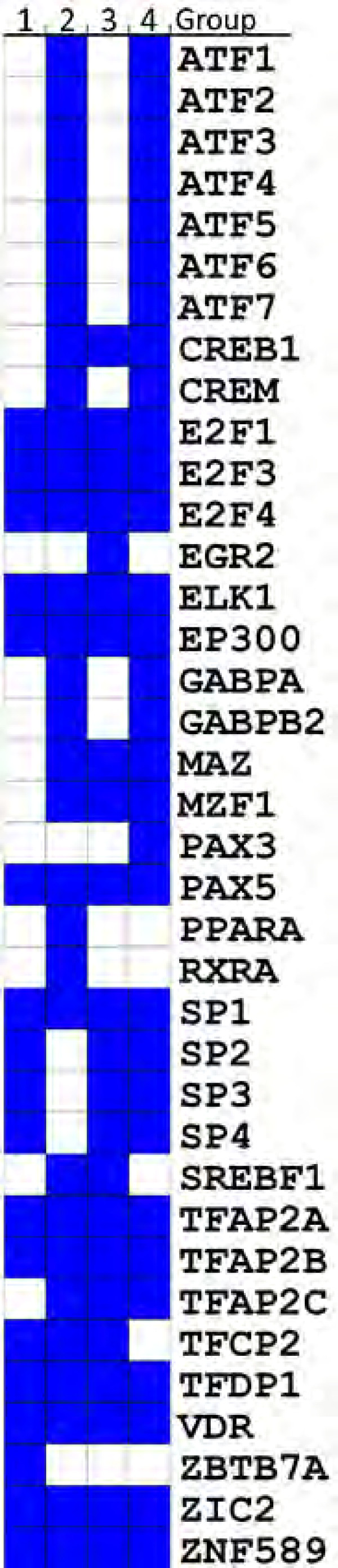
**Heat-map showing combinations of TFs regulating different sets of genes**. Blue blocks across the groups (columns) indicate that several TFs (rows) may potentially regulate genes in different groups.

The enriched TFBSs (i.e. ORI > = 2, Materials and Methods) were used for network construction. The TFs potentially regulating maximum number of genes in each group were identified. The TFs were ranked based on the number of genes they regulate as demonstrated via TFBSs on the promoters of the genes. We used a cut-off of 80^th ^percentile to identify the TFs regulating maximum number of genes in a group. Any TF having TFBSs in genes below 80^th ^percentile were not included in further analysis. These steps lead us to identify TFs unique to sets of genes in each group (Figure 3). Figure 3 also show that multiple TFs may regulate set of genes in different groups thus highlighting the TF families that could be of relevance for further investigations in OC.

Using the cut-off as mentioned above, we identified 18 TFs having TFBSs in promoters of 11 genes in group 1. The 54 genes in group 2 contained TFBSs for 31 TFs. In group 3, we found TFBSs for 23 TFs in promoters of 66 genes. For group 4, we identified TFBSs for 31 TFs in promoters of 192 genes. We then combined groups 1 and 3 because the genes in these groups were found experimentally to be controlled by estrogen. This new group we refer to as 'experimentally controlled genes'. We found that there were 17 TFs common to both gene groups 1 and 3, with only one TF unique to group 1 and six TFs unique to group 3 (additional file 3). Thus, we found 24 TFs that have TFBSs on the promoters of experimentally controlled genes only. The comparison of these 24 TFs with group 2 (group of genes containing predicted EREs but lacking experimental evidence of control by estrogen) revealed that there were 19 TFs common with the experimentally controlled genes, and 12 TFs unique to group 2 (additional file 4). Similar comparison of TFs for experimentally controlled genes with group 4 revealed 20 common TFs and 11 unique TFs for group 4 (additional file 5).

The above analysis suggests that combinations of TFs could regulate different sets of genes (Figure 3). We propose that the genes in group 2 can be considered as new targets for experimental evaluation of estrogen control. This proposition is based on the presence of predicted EREs in promoters of these genes. The presence of EREs in promoters of genes does not establish that the genes are under estrogen control, however, the ERE predictions open a possibility that these genes may be under such a control.

#### (iv) Identification of potential markers for 323 OC genes

We propose that the TFs regulating a maximum number of OC genes can be regarded as potential candidates for biomarkers for OC. This is based on the concept that such TFs are the drivers of the potential deregulation of genes in OC, and thus are relevant for use as biomarkers. To identify potential candidates, we ranked TFs with regards to the number of genes that have TFBSs for a particular TF (see additional file 6 for list of all ranked TFs). The ranking produced a list of 17 TFs (Table 1) each potentially regulating more than 200 OC genes. Top ranked three TFs (entrez ID 5079, 7020 and 1385) had TFBSs in promoters of 289, 268 and 255 genes, respectively. The top ranked TF (PAX-5) belongs to a family of paired box TFs. The protein product of this gene is a B-cell lineage specific activator and has been identified as a marker for the discrimination of low- to intermediate-grade pulmonary neuroendocrine carcinomas from high-grade with 100% specificity and 79% sensitivity in surgical specimens [37] and for diagnosis of undifferentiated malignant neoplasms [38]. The TF ranked at position two in the list (entrez ID 7020) is AP-2 and regulates the expression of amyloid precursor protein in oral squamous cell carcinomas [39]. It has also been linked with breast cancer [40]. The third ranked TF (entrez ID 7546) is zinc finger protein (ZIC2) and has been found to be differentially expressed in endometrial cancers with lymph node metastasis [41] and has even been patented as a small-cell lung cancer associated antigen (US Patent 7314721, issued on January 1, 2008, http://www.patentstorm.us/patents/7314721/fulltext.html.

**Table 1:** Rankings of TFs (potential biomarkers) for complete gene set of 323 OC genes and for the 'experimental group'

Ranked TFs for 323 genes				Ranked TFs for Experimental group			
**TF_ID**	**TF_symbol**	**Number of Gene Targets** **(out-degree)**	**Ranks**	**TF_ID**	**TF_symbol**	**Number of Gene Targets** **(out-degree)**	**Ranks**

5079	PAX5	289	1	5079	PAX5	68	1

7020	TFAP2A	268	2	7020	TFAP2A	60	2

***1385***	***CREB1***	*255*	**3**	7546	ZIC2	60	3

7546	ZIC2	253	4	1869	E2F1	57	4

7421	VDR	249	5	51385	ZNF589	55	5

1869	E2F1	248	6	7027	TFDP1	54	6

***7022***	***TFAP2G***	*246*	**7**	1871	E2F3	54	7

7027	TFDP1	245	8	7421	VDR	54	8

1874	E2F4	245	9	1874	E2F4	54	9

1871	E2F3	245	10	7021	TFAP2B	52	10

7021	TFAP2B	242	11	6670	SP3	52	11

2033	EP300	223	12	2033	EP300	52	12

6670	SP3	222	13	6667	SP1	48	13

6667	SP1	216	14	2002	ELK1	48	14

51385	ZNF589	216	15	***7024***	***TFCP2***	***42***	15

***7593***	***ZNF42***	*214*	16	***6668***	***SP2***	***40***	16

2002	ELK1	210	17	***6671***	***SP4***	***40***	17

TFs unique to both groups are shown in bold italics.

#### (v) Identification of potential markers for estrogen regulated genes

After identifying TFs potentially regulating a maximum number of OC genes, we looked for TFs uniquely regulating estrogen controlled genes in groups 1 and 3. For the 'experimentally controlled genes', we identified 17 top ranked TFs (Table 1). This group has three unique TFs ranked 15, 16 and 17, while others were common with the previous ranked list of TFs (for all categories combined). The TF ranked at position 15 is CP2 (entrez ID 7024), which has been targeted as a diagnostic marker for ovarian carcinoma [42]. The combination of CA125 with CP2 has been reported to be sensitive in diagnosing non-mucinous ovarian tumors [43]. The TFs ranked 16 and 17 in the list are Sp2 (entrez ID 668) and Sp4 (entrez ID 6671), respectively. The PubMed search using keywords "Sp4 ovarian cancer" retrieved only one abstract related to the study that tested new analogues of a natural antibiotic 'Mithramycin' as inhibitors of Sp1-dependent transcription in OC xenografts [44]. Therefore, we propose that Sp4 can be investigated as a potential new gene or marker for OC. Another observation is that the family of Sp TFs might have an important role to play in OC. Sp3 and Sp4 are involved in estrogen mediated gene expression in MCF-7 breast cancer cells [45]. Sp3 (entrez ID 6670) has been shown to control the expression of *PTEN *(phosphatase and tensin homolog deleted on chromosome 10) in ovarian carcinomas [46]. The proximal binding of Sp1 to the TSS has been demonstrated to be a requirement for positive interactions with the ER to enhance transcription and also to stabilize weak interactions of ER [47]. Three TFs (TFCP2, SP2 and SP4) unique to estrogen controlled group can be tested as specific biomarkers for the sub-group of patients that are influenced by estrogen hormones.

The analysis presented above has enabled us to identify potential biomarkers that putatively regulate transcription of a group of OC-associated genes. The results presented here can be justified by the fact that the analysis could identify some of the known biomarkers (currently under investigation), such as CP2 and ZIC2. The study has identified unique potential biomarkers for a sub-group of genes that are known to be under estrogen control.

### (c) Preliminary validation of biomarkers using publically available data

To see if the TFs identified in the current study have functional roles and the potential to be used as diagnostic biomarkers during early and advanced stages of OC, we looked at genes specifically (over-expressed) as represented in microarray analysis at different stages of ovarian serous adenocarcinoma and metastatic ovarian serous papillary adenocarcinoma tissue samples [48]. For identifying the genes and potential TFs associated with these genes we used data from 24 non-metastatic ovarian cancer tissue samples against 3 metastatic ovarian cancer tissue samples (obtained from microarray gene expression database) CleanEx (Microarray database) http://www.cleanex.isb-sib.ch/index.html[49] using dataset number AFFY004 [50]. The primary data was used in dataset number AFFY004 was taken from [48] and the details of primary data have been summarized (additional file 7). We could identify three TFs (*ZIC2, E2F4 *and *TFCP2*) in this dataset of over-expressed genes in OC. TFs such as *E2F1, E2F3, TFAP2A, VDR, TFAP2G, SP3, ZNF42, CREB1 *and *ELK1 *were found to be over-expressed by > = 1.5 folds in OC datasets analyzed in Oncomine http://www.oncomine.org/[51]. Our study has shown that 65% of the TFs regulating genes in a full set of 323 OC genes and 47% of the TFs regulating estrogen controlled sub-set of genes of our predication set could be identified in real-time data from published microarray expression studies (Table 2). This analysis shows that the expression of a number of the TFs identified in the present study are affected in OC and these TFs may have the potential to be used as biomarkers. By definition, a biomarker is useful if it can detect the disease in early stages in a patient as compared to a healthy individual. To study this, we performed an analysis based on the expression profiles available in Oncomine targeting 'Ovarian serous adenocarcinoma' which is the most common form of the OC [52-54]. We could identify *TFAP2A, VDR, TFAP2G *and *E2F3 *as over-expressing genes in at least one of the three datasets we studied (Table 3). The expression of *CA125 *(a known biomarker of OC) was not identified in one of the datasets under investigation [52].

**Table 2:** Validation of biomarkers based on over-expression in published OC microarray datasets available in databases such as CleanEx and Oncomine

	TFs regulating all 323 genes		TFs regulating Estrogen controlled sub-set of genes	
	**Out of top ranked 17**		**Out of top ranked 17**	

**CleanEX**	**1**		**2**	

Oncomine	Fold change > = 2	Fold change > = 1.5	Fold change > = 2	Fold change > = 1.5

TFs found	8	10	6	6

**Total over-expressed TFs**	**9**	**11**	**8**	**8**

**Table 3:** The average fold-change in expression of TFs identified as potential biomarkers in the present study and CA 125 (a known OC biomarker) in ovarian serous adenocarcinoma tissues samples in comparison to normal tissue based on three OC pre-analyzed datasets available in Oncomine

Biomarkers	**Lu et al., 2004 **[54]	**Hendrix et al., 2006 **[53]	**Adib et al., 2004 **[52]
**TFAP2A**	1.99	1.57	1.99

**VDR**	1.66	1.21	1.22

**TFAP2G**	1.33	1.85	2.18

**E2F3**	1.84	1.18	2.65

**CA 125**	3.33	2.97	--

The values in the table represent the fold-change of expression of genes with p-values < 0.05 and the gene ranks in top 10% (pre-calculated in Oncomine).

**Figure 4:**
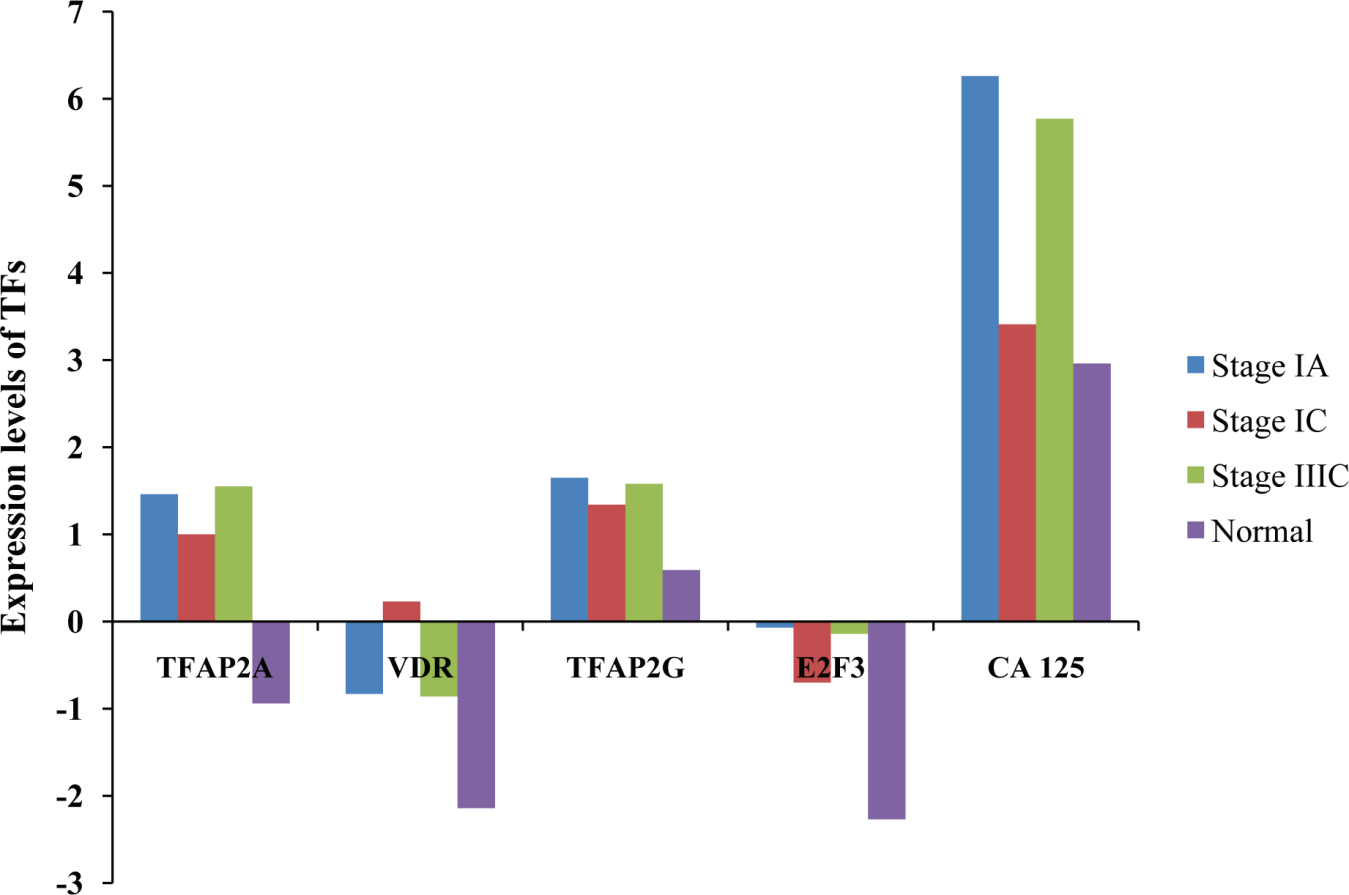
**The comparison of the expression levels of TFs identified as potential biomarkers in the present study and CA 125 (a known OC biomarker) in randomly chosen ovarian serous adenocarcinoma tissue samples categorized at stages IA, IC and IIIC in comparison to normal tissue based on OC pre-analyzed dataset of study by Lu et al., 2004 **[54]**available in Oncomine**.

The next step of the investigation involved in-depth analysis of expression of above TFs at different stages of OC. For this, we compared the expression levels of genes in cancerous tissues categorized as stage IA, IC and IIIC [54]. Figure 4 explains that the expression of *TFAP2A, VDR, TFAP2G *and *E2F3 *genes is higher in early stages (stages IA and IC) as well as advanced stage (IIIC) of the OC as compared to the normal tissue. The same applies to the gene expression levels of *CA125*. This analysis clearly shows that the proposed TFs can be tested as diagnostic biomarkers for OC in detailed laboratory investigations. Other important information linked to some of the TFs that are proposed as biomarkers is their detectable expression in blood. For example, VDR [55], E2F3 [56] and CREB1 [57] can be easily detected in the blood and blood-based diagnostic assays can be easily developed to measure these TFs if validated as biomarkers of OC. Further experimental investigations can also focus on establishing a link between blood concentration of biomarkers and pathological state of the ovary in OC.

Since the biomarkers identified were found to be promising we further looked at the pathways involved/associated with the 17 TFs. The current data demonstrates that the TFs identified as biomarkers are associated with cell cycle and cell signaling pathways that were known to have a significant role in affecting cancer at the initiation stage as well as metastatic spread (additional files 8 and 9). Functional categorization as identified using Gene/Gene Set overlap matrix with 17 TFs identified as biomarkers for full set of OC genes (additional file 8) showed overlap which were prominent for cell cycle associated TFs based on data from *E2F1, E2F3-4 *and *TFDP-1 *genes. Estrogen- controlled sub-set of OC genes were also found to be under the influence of cell cycle associated TFs (additional file 9). Overlap pattern indicates that TFs associated with G1 to S phase transition were found to be mostly affected. Transcription factors DP-1 and E2F1 showed similar overlap patterns in the gene sets since *TFDP-1 *encodes a member of a family of transcription factors that heterodimerize with E2F proteins to enhance their DNA-binding activity and promote transcription from E2F target genes.

We propose that the TFs identified as biomarkers in the current study may have a prominent role associated with genes involved in progression of OC. Our previous study has identified E2F5 (a member of E2F family of TFs) as a potential marker for detecting the malignancy associated with OC [6].

### (d) Physiological significance of predicted TFs

Here we describe the significance of TFs identified in our current study and relevance of these TFs to OC as reported by recent studies. There is growing evidence that deregulation of cell cycle associated transcription factors of E2F family (E2F1 to E2F8) is causatively involved in the patho-physiology of various tumors. Proliferation-promoting E2F transcription factors, E2F1 and especially E2F2 play a pivotal role in tumor biology of OC and may be candidates for specific therapeutic targets [58]. In the same study, authors demonstrate higher expression of E2F family of TFs in 77 ovarian cancer samples except E2F6. They also suggested that expression levels of E2F1 and E2F2 are associated with highly malignant and fast growing tumors. Deregulation of both proliferation-promoting and proliferation-inhibiting E2F TFs and their cross-talk were reported to influence the clinical outcome Therefore; appearance of three members of E2F family in our top ranked list also warrants further investigations to explore biomarker potential of these TFs. CREB1 is another TF identified in the current study is associated with *MMP-2 *transcription, which is involved in pro-metastatic function along with *TG-2*. EP-300 protein is a histone acetyltransferase and regulates transcription via chromatin remodeling. It is found that this protein regulates important cellular processes such as cell proliferation and differentiation. The putative tumour suppressor gene *EP300 *is located on the region 22q13 that shows frequent loss of heterozygosity (LOH) in colon, breast and ovarian cancers. In epithelial cancers, *EP300 *is mutated and provide the evidence that it behaves as a classical tumor-suppressor gene [59]. Higher immunoreactivity of vitamin D receptor (VDR) was found in breast, ovarian and cervical carcinomas as compared to normal corresponding tissues [60]. Recently, the *VDR *polymorphism FokI was shown to be associated with susceptibility to OC. These results suggest that the *VDR *polymorphisms from the FokI genotype may be associated with improved prognosis of patients with EOC [61]. The *PAX5 *gene belongs to the paired box (PAX) family of transcription factors. PAX5 has been reported to be present in ovarian tumor tissues and plasma [62]. Elk-1 has been associated with OC progression [63].

One should observe the following limitations of our methods. We have several constraining factors involved. First, we limited consideration of computational analysis of transcription regulation to promoters of limited size proximal to 5' gene ends. While there is no method to accurately pinpoint boundaries of promoter regions, we used 1000 bp upstream and 200 bp downstream of TSS as an approximation of the actual promoters. Second, a lot of gene control is exerted from the remote control regions such as enhancers and silencers. Since we had no means to determine these regions for individual genes we did not consider such regulatory effects. Thirdly, we limited our consideration to TFs that fall above 80^th ^percentile regarding the number of genes they potentially regulate in OC gene set we considered. Finally, the set of genes implicated in OC that we used is by no means complete. However, in spite all these practical odds, the method produced results that are in high concordance with the known biological facts (a number of the top ranked TF are either already known diagnostic markers of represent previously proposed markers for diagnosis of OC) and also implies some of the new diagnostic markers for OC.

## Conclusions

The analysis presented here has generated potential molecular targets for evaluation as possible players in the causation or characterization of OC. The ranked lists of TFs can be used to prioritize putative biomarkers based on their potential to regulate a large number of genes implicated in OC. The analysis shown here has provided deeper insights into the transcriptional regulation of many genes involved in OC. We highlight that this is an understudied field and the use of bioinformatics tools could enhance our insights into the underlying genetic mechanism of OC. This study has provided a list of potential target genes that could be tested in the laboratory and ultimately be targets for therapeutic treatments.

## Methods

### Outline of methodology

The study identified several genetic targets for evaluation as potential diagnostic markers of OC. The promoters of 323 genes implicated in OC [64] were screened for EREs (estrogen response elements). To identify the genes experimentally validated as estrogen responsive, we cross-checked against two databases of documented estrogen-responsive genes, namely KBERG [32] and ERTargetDB [33], neither of which is comprehensive. We divided the genes into four groups: 1) those having predicted EREs and experimental evidence of estrogen control; 2) have predicted EREs but no experimental evidence of estrogen control, 3) have no predicted EREs but have experimental evidence of estrogen control, and 4) neither predicted EREs nor experimental evidence of estrogen control. We then predicted TFBSs (Transcription factor binding sites) on the promoters of OC genes so as to provide a link between genes and TFs that regulate them. For this purpose the Transfac Professional database ver. 11.4 [65] was used. The TFBSs were mapped to the promoters of OC genes using the Match program [66] of the Transfac suite. The enrichment of the mapped TFBSs in the target promoter set relative to a background set was determined. The enriched TFBSs were associated with TFs that are predicted to bind them. In this way we established a link between the target genes and the TFs that regulate their expression. This data was used to reconstruct parts of the transcription regulatory networks of relevance to OC. These networks were analyzed to identify crucial regulators of gene expression, opening the way to identify potential biomarkers that characterize OC gene transcription.

### Identification of genes implicated in OC

The genes implicated in OC were determined as follows. Initially a list of 900 genes was collected from available online resources like Cancer Gene Census [67]http://www.sanger.ac.uk/genetics/CGP/Census/, GeneCards [68]http://www.genecards.org/index.shtml, SymAtlas [69], OMIM [70] (Online Mendelian Inheritance in Man, 2007) http://www.ncbi.nlm.nih.gov, Ovarian Kaleidoscope Database [71]http://ovary.stanford.edu, Entrez Gene [72]http://www.ncbi.nlm.nih.gov/sites/entrez?db=gene and GenAtlas [73]http://www.genatlas.org/. This list was reduced to 379 by biologists after carefully going through the relevant literature. The genes included in the study were filtered through a strict criterion that the gene expression must be experimentally confirmed in either OC tissue using one of the techniques such as: RT-PCR, immunohistochemistry, western blotting or FISH (Fluorescent In Situ Hybridization). If a gene is documented as having an OC linked SNP, it was also included. The collection of these 379 genes can be explored using DDOC database http://apps.sanbi.ac.za/ddoc/[64].

### Promoter sets

The promoter sequences determined as a region covering [-1000, +200] relative to the transcription start sites (TSS) at the 5' ends of genes were extracted from the Fantom3 dataset [74,75]. The promoters were extracted for 323 Entrez IDs corresponding to the OC genes. As a background set, we used a set of selected 11,000 sequences of length 1200 bases from human genome.

### Prediction of Estrogen Response Elements (EREs)

The tool Dragon ERE Finder [76] version 6.0 http://apps.sanbi.ac.za/ere/index.php was used to predict the EREs on the promoters.

### Matching to genes from KBERG database

The OC genes were cross-checked against all the 1516 experimentally confirmed estrogen-responsive genes in the KBERG database [32].

### Matching to genes in ERTargetDB database

The OC genes were cross-checked against the ERTargetDB [33], http://bioinformatics.wistar.upenn.edu/ERTargetDB, which contains:- (a) 40 genes with 48 experimentally verified ERE direct binding sites and 11 experimentally verified ERE tethering sites; (b) 42 genes identified via ChIP-on-chip assay for estrogen binding (c) 355 genes from gene expression microarrays; (d) 2659 computationally predicted genes.

### Transcription Factor binding sites (TFBSs) mapping

TFBSs were mapped to promoter sequences using all mammalian matrix models of TFBSs contained in the TRANSFAC Professional database v.11.4 [65]. For this purpose, we used the Match program with a *minFP *profile for the threshold of the matrix models since the minFP profile contains the optimized threshold values for the core and matrix scores [66] that provide minimum presence of false positive predictions in the predicted TFBS set.

### Enrichment of TFBSs patterns and edge identification

Enrichment of TFBSs found in the target set was determined using the method from [77,78]. The mapped TFBSs were ranked based on their over-representation index (ORI) [77] and those that were sufficiently enriched (ORI > = 2, as determined by [77]) were used for further analysis. This step produced links between the target genes and TFs that regulate their expression and represent an edge for network reconstruction.

### Network reconstruction and identification of biomarkers

The genes were clustered again based on the promoter content (presence of TFBSs) and this allowed us to identify the network of genes potentially regulated by individual TFs or their combinations. The identified TFs were ranked based on the number of genes they regulate above 80^th ^percentile. Finally, a list of ranked TFs was generated.

### Functional and pathway analyses of network genes

After identification of networks of genes, next step was to classify these genes based upon their functional properties. We used the existing gene ontology (GO) annotation [79] information for the genes. GO term analysis was performed for all the genes using DAVID (The **D**atabase for **A**nnotation, **V**isualization and **I**ntegrated **D**iscovery) version 2.0 [80] and transcripts were clustered based on their functional annotations at GO level 4. This helped us to identify the functional clustering of the genes and their role as a group in various biological processes.

Functional analysis is very useful but provides limited information regarding the involvement of genes in specific pathways. The gene lists were mapped to KEGG [81] pathways using DAVID. Mapping of target genes to KEGG helped us to identify pathways critical to the etiology and development of OC.

The above described methodology enabled us to identify networks of genes potentially regulated by similar TFs and their possible roles in etiology and development of OC, opening a way for insights that could lead to the identification of potential diagnostic markers of OC.

## Abbreviations

OC: Ovarian cancer; ERα: estrogen receptor alpha; ERs: estrogen receptors; HREs: Hormone-Response Elements; EREs: estrogen response elements; AP: activator protein; *NF-ß*: nerve factor-ß; TFs: Transcription factors; TFBSs: TF binding sites; TSSs: transcription start sites; *PTEN *: phosphatase and tensin homolog deleted on chromosome 10; LOH: loss of heterozygosity; *VDR*: vitamin D receptor; FISH: Fluorescent In Situ Hybridization; ORI: over-representation index; GO: gene ontology.

## Authors' contributions

MK and VBB conceptualized and designed the study, performed analysis, interpreted data, wrote and revised the manuscript. CMP and SS analyzed the data. KN and MC participated in validation of the data and assessed the biological significance of the predictions. All authors have given final approval of the version to be published.

## Conflicts of interest

The authors declare that they have no competing interests.

## Supplementary Material

Additional file 1 **Functional analysis of all four groups of genes using DAVID**. Pathway analysis performed on all four groups of genes using DAVID. Click here for file

Additional file 2 **Distribution of TFBSs and EREs in promoters of genes that contain predicted EREs**. Details of gene IDs and positions on promoters where TFBSs and EREs were predicted to have binding sites. Click here for file

Additional file 3 **TFs common and unique to gene groups 1 and 3**. This file contains list of genes that were found to be common and unique to groups 1 and 3. Click here for file

Additional file 4 **TFs common and unique to gene groups 'experimentally controlled genes' and 3**. This file contains list of genes that were found to be common and unique to groups 3 and 'experimentally controlled genes'. Click here for file

Additional file 5 **TFs common and unique to gene groups 'experimentally controlled genes' and 4**. This file contains list of genes that were found to be common and unique to groups 4 and 'experimentally controlled genes'. Click here for file

Additional file 6 **TFs with ranks for a set of 323 genes**. The list of all TFs with ranks based on number of genes they potentially control from full set of 323 genes. Click here for file

Additional file 7 **Details of primary source of the data for partly validating the biomarkers using CleanEx database**. Description of previously published study material that was used for partial validation of biomarkers identified in the current study. Click here for file

Additional file 8 **Functional categorization as identified using Gene/Gene Set overlap matrix with 17 TFs identified as biomarkers for full set of OC genes**. The graphical view is a matrix of collections of gene sets, where each colored entry indicates that the two gene sets have a statistically significant overlap. Overlap between gene and gene sets were prominent for cell cycle associated TFs as observed for the first two rows from E2F1, E2F3-4 and TFDP-1 genes). Functional relevance of top ranked 17 biomarkers identified for full set of 323 OC genes in the present investigation. Click here for file

Additional file 9 **Functional categorization as identified using Gene/Gene Set overlap matrix with 17 TFs identified as biomarkers for estrogen-controlled sub-set of 77 OC genes**. The graphical view is a matrix of collections of gene sets, where each colored entry indicates that the two gene sets have a statistically significant overlap. Overlap between gene and gene sets were prominent for cell cycle associated TFs. Functional relevance of top ranked 17 biomarkers identified for estrogen-controlled sub-set of 77 OC genes in the present investigation. Click here for file
