# Supplementary material for: In Silico discovery of transcription factors as potential diagnostic biomarkers of ovarian cancer
Source: BMC Syst Biol. 2011 Sep 19;5:144. doi: 10.1186/1752-0509-5-144 (PMC3184078; doi:10.1186/1752-0509-5-144)
Supplement: Additional file 7 — Details of primary source of the data for partly validating the biomarkers using CleanEx database. Description of previously published study material that was used for partial validation of biomarkers identified in the current study. [file 1752-0509-5-144-S7.DOCX]

**Primary source of the data for partly validating the biomarkers was obtained from the following reference.**

Welsh JB, Zarrinkar PP, Sapinoso LM, Kern SG, Behling CA, Monk BJ, Lockhart DJ, Burger RA, Hampton GM. Analysis of gene expression profiles in normal and neoplastic ovarian tissue samples identifies candidate molecular markers of epithelial ovarian cancer. Proc Natl Acad Sci U S A. 2001 Jan 30;98(3):1176-81.

**Sample information used for generation microarray data**

The data for the current study is obtained from the manuscript published by Welsh et al., 2001. The data was found appropriate since the data represent the serous papillary type of adenocarcinoma which is a very common type among ovarian cancer and is often associated with aggressiveness among the solid epithelial tumor affecting the ovary. The authors (Welsh et al., 2001) have used samples that were selected specifically to analyse the gene expression profiles in normal and neoplastic ovarian tissue samples to identify candidate molecular markers of epithelial ovarian cancer. For this purpose the authors used twenty-seven flash-frozen serous papillary adenocarcinomas of the ovary and three normal samples of whole ovarian tissue were made available through the Cooperative Human Tissue Network (CHTN Midwestern Division, Columbus, OH). The detailed clinical and pathologic details of the samples were tabulated in supplementary Table 1 as reported by Welsh et al., (2001), which were published as supplemental data on the PNAS web site, [www.pnas.org](http://www.pnas.org). Each tumor was histologically analyzed. For generating gene expression data the authors have used oligonucleotide microarrays with probe-sets complementary to more than 6,000 human genes to monitor the levels of expression within aggregate normal and malignant ovarian tissues. Data obtained from normal and malignant ovarian tissues made it possible to distinguish the two classes of tissue based on quantitative expression levels. The authors have described the expression profiles of different tumours to demonstrate significant heterogeneity, but mentions that it is nonetheless possible to identify groups of genes whose altered expression may influence their clinical behaviour.

**Primary results: Assessment for the primary data**

To identify patterns of gene expression in normal and malignant ovarian tissue the authors have used cluster analysis based on hybridization intensity values for each gene. A total of 1,243 genes with average hybridization intensities > 0 that varied most across the samples (SD ≥250), were used for identifying genes with strong differential expression. Their study has resulted in the identification of Clusters of Genes Expressed in Normal and Malignant Tissues. The tight clustering of the normal tissues was largely ‘‘driven’’ by two distinct profiles of gene expression (1) The first was represented by a group of about 100 genes that were highly expressed in normal tissue and underexpressed in the tumors and cell lines (2) the second profile was represented by several smaller clusters of genes that were underexpressed in normal tissue as compared with ovarian tumors. For detailed description of the primary analyses please refer to the results section by Welsh et al., (2001).
