# Supplementary figures and images for: In Silico discovery of transcription factors as potential diagnostic biomarkers of ovarian cancer
Source: BMC Syst Biol. 2011 Sep 19;5:144. doi: 10.1186/1752-0509-5-144 (PMC3184078; doi:10.1186/1752-0509-5-144)

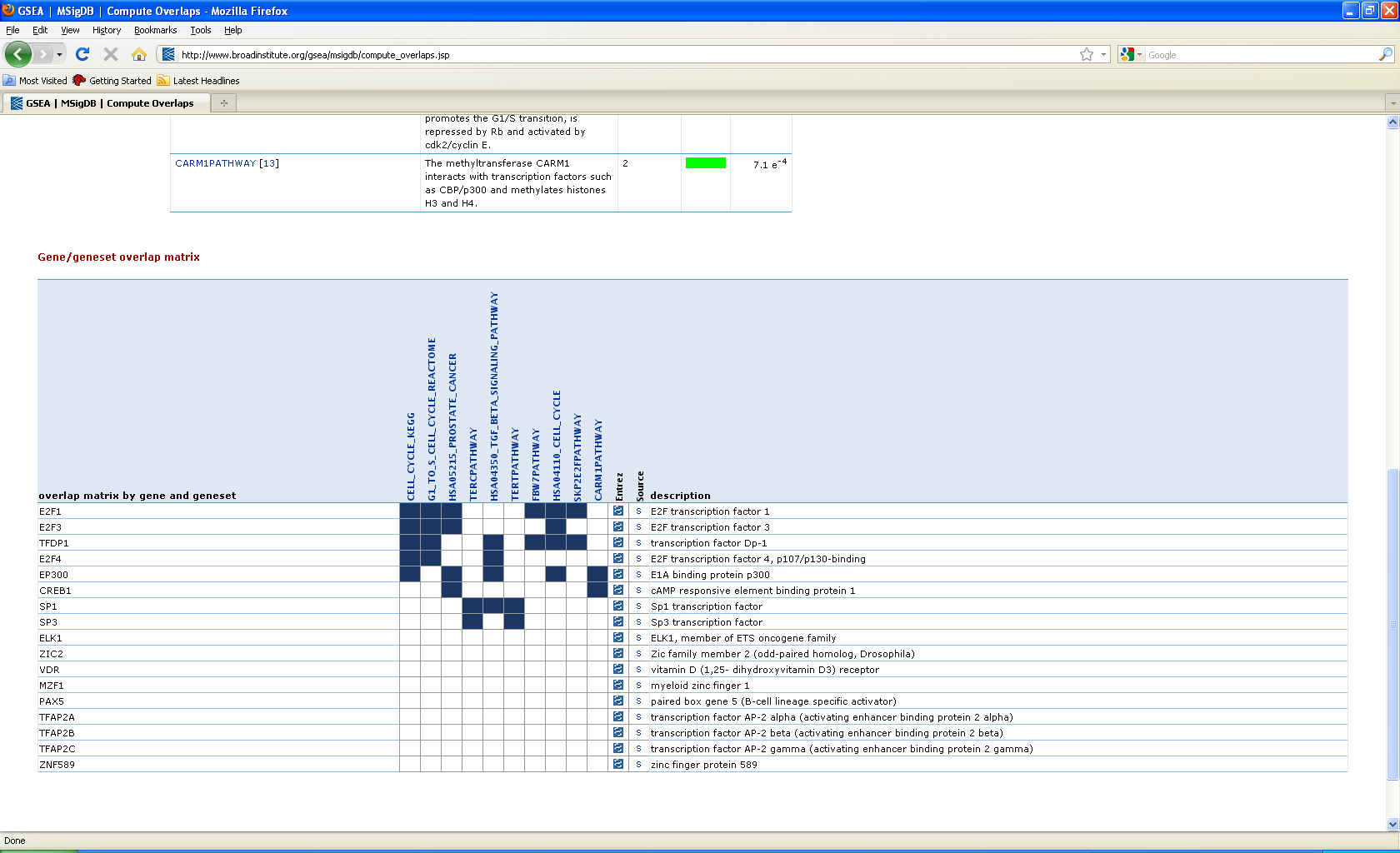

Supplement: Additional file 8 — Functional categorization as identified using Gene/Gene Set overlap matrix with 17 TFs identified as biomarkers for full set of OC genes. The graphical view is a matrix of collections of gene sets, where each colored entry indicates that the two gene sets have a statistically significant overlap. Overlap between gene and gene sets were prominent for cell cycle associated TFs as observed for the first two rows from E2F1, E2F3-4 and TFDP-1 genes). Functional relevance of top ranked 17 biomarkers identified for full set of 323 OC genes in the present investigation. [file 1752-0509-5-144-S8.DOCX]

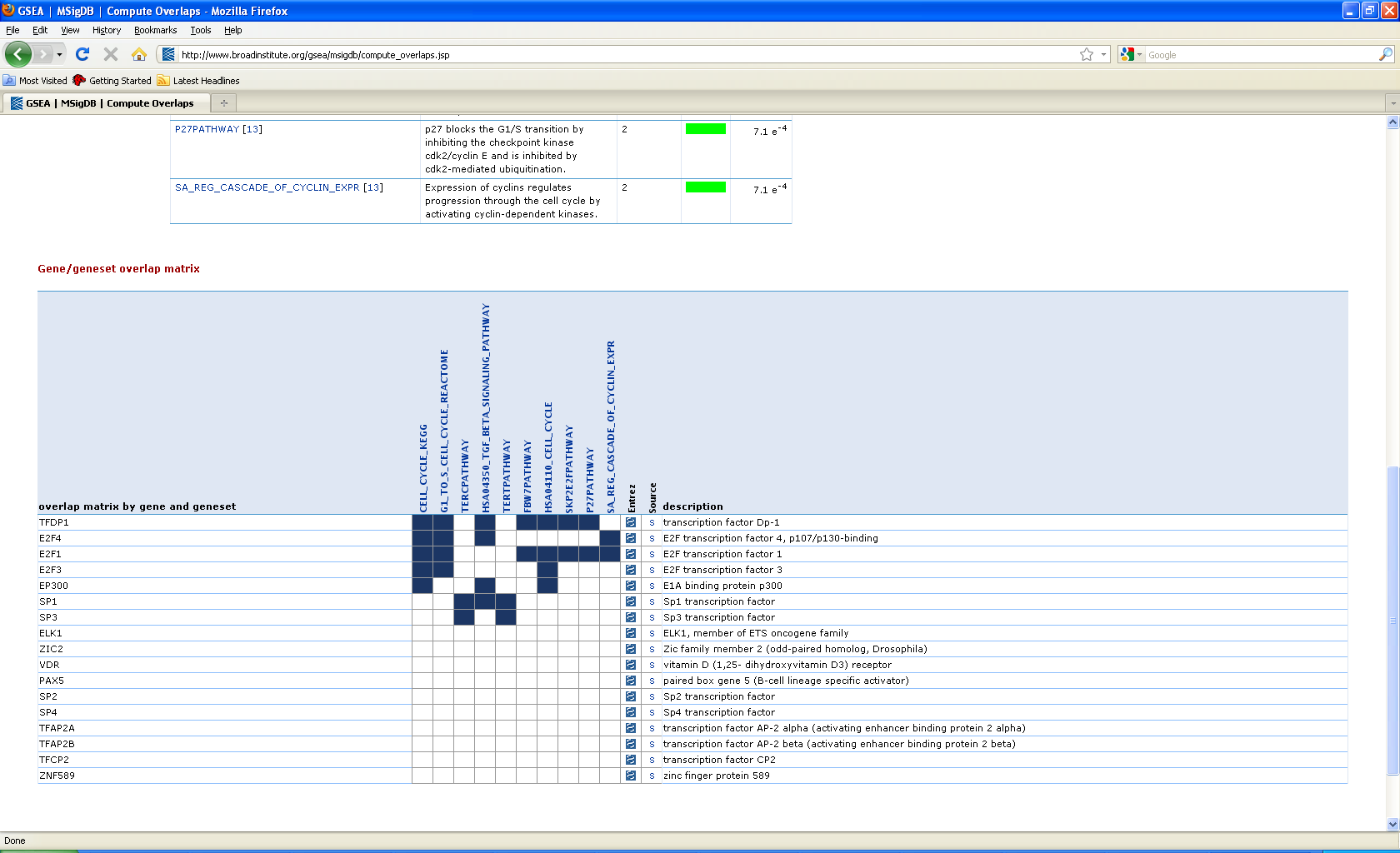

Supplement: Additional file 9 — Functional categorization as identified using Gene/Gene Set overlap matrix with 17 TFs identified as biomarkers for estrogen-controlled sub-set of 77 OC genes. The graphical view is a matrix of collections of gene sets, where each colored entry indicates that the two gene sets have a statistically significant overlap. Overlap between gene and gene sets were prominent for cell cycle associated TFs. Functional relevance of top ranked 17 biomarkers identified for estrogen-controlled sub-set of 77 OC genes in the present investigation. [file 1752-0509-5-144-S9.DOCX]
